# Laryngeal Lesion Classification Based on Vascular Patterns in Contact Endoscopy and Narrow Band Imaging: Manual Versus Automatic Approach

**DOI:** 10.3390/s20144018

**Published:** 2020-07-19

**Authors:** Nazila Esmaeili, Alfredo Illanes, Axel Boese, Nikolaos Davaris, Christoph Arens, Nassir Navab, Michael Friebe

**Affiliations:** 1 INKA-Application Driven Research, Otto-von-Guericke University Magdeburg, 39120 Magdeburg, Germany; alfredo.illanes@med.ovgu.de (A.I.); axel.boese@ovgu.de (A.B.); michael.friebe@ovgu.de (M.F.); 2 Department of Otorhinolaryngology, Head and Neck Surgery, Magdeburg University Hospital, 39120 Magdeburg, Germany; nikolaos.davaris@med.ovgu.de (N.D.); christoph.arens@med.ovgu.de (C.A.); 3 Chair for Computer Aided Medical Procedures and Augmented Reality, Technical University Munich, 85748 Munich, Germany; navab@cs.tum.edu; 4 IDTM GmbH, 45657 Recklinghausen, Germany

**Keywords:** laryngeal cancer, contact endoscopy, narrow band imaging, automatic classification, feature extraction, machine learning

## Abstract

Longitudinal and perpendicular changes in the vocal fold’s blood vessels are associated with the development of benign and malignant laryngeal lesions. The combination of Contact Endoscopy (CE) and Narrow Band Imaging (NBI) can provide intraoperative real-time visualization of the vascular changes in the laryngeal mucosa. However, the visual evaluation of vascular patterns in CE-NBI images is challenging and highly depends on the clinicians’ experience. The current study aims to evaluate and compare the performance of a manual and an automatic approach for laryngeal lesion’s classification based on vascular patterns in CE-NBI images. In the manual approach, six observers visually evaluated a series of CE+NBI images that belong to a patient and then classified the patient as benign or malignant. For the automatic classification, an algorithm based on characterizing the level of the vessel’s disorder in combination with four supervised classifiers was used to classify CE-NBI images. The results showed that the manual approach’s subjective evaluation could be reduced by using a computer-based approach. Moreover, the automatic approach showed the potential to work as an assistant system in case of disagreements among clinicians and to reduce the manual approach’s misclassification issue.

## 1. Introduction

Laryngeal cancer is the second most frequent malignant tumor of the head and neck region [1]. The vast majority of primary laryngeal cancers are Squamous Cell Carcinomas (SCC) arising from the epithelial lining of the larynx, mostly as a result of tobacco and alcohol consumption. A total of 40% of these cancers are diagnosed at an advanced stage, which is associated with a poorer prognosis and quality of life [2]. The early diagnosis of laryngeal cancer is crucial to reduce patient mortality and preserve vocal fold function.

Specific changes in the morphology and three-dimensional orientation of the vocal fold’s sub-epithelial blood vessels have proved to be associated with the development of benign and malignant laryngeal lesions. Several approaches have been proposed to describe and classify these vascular changes. Among the complex classification systems proposed by [3] and [4], the European Laryngological Society (ELS) introduced a simplified classification that divides vascular changes into longitudinal and perpendicular classes [5,6]. Longitudinal Vascular Changes (LVC) spread along the length and width of the vocal fold and can be observed in all kinds of benign or malignant lesions. On the contrary, Perpendicular Vascular Changes (PVC) develop perpendicularly towards the mucosa, as a result of neoangiogenesis in laryngeal Papillomatosis, pre-malignant and malignant histopathologies.

The endoscopic detection and evaluation of vascular changes can provide complementary diagnostic information for clinicians to detect and differentiate between benign and malignant laryngeal lesions [7]. As a minimally-invasive endoscopic technique, Contact Endoscopy (CE) can provide real-time visualization of cellular and vascular structures of the laryngeal mucosa [8,9]. For the purpose of detecting and evaluating superficial vascular changes, several enhanced endoscopic techniques such as Narrow Band Imaging (NBI) have been combined with CE to ease the detection of vascular changes [10]. The use of enhanced CE showed promising results in the assessment of vascular patterns followed by indicative of various laryngeal pathologies [4,11,12].

Clinicians can receive useful information about the type and suspected histopathology of laryngeal lesions by evaluating LVC and PVC in enhanced CE images; however, it is a challenging task for them. There are similarities between vascular patterns of benign and malignant laryngeal lesions. The PVC with wide-angled turning points, as observed in laryngeal Papillomatosis can be difficult to distinguish from PVC with narrow-angled turning points, as observed in pre-malignant and malignant histopathologies [5,12,13,14]. Hence, the interpretation of vascular patterns in enhanced CE images requires an extensive learning curve from the clinicians to reduce the risk of subjective evaluation that can cause potential problems in differentiation between benign and malignant laryngeal lesions [4,10,12,15,16].

In this study, we first aimed to present the results of manual versus automatic classification of benign and malignant laryngeal lesions based on the vascular patterns in CE-NBI images. We then evaluated the issues of manual classification and subsequently showed how a computer-based approach can assist the clinicians to overcome these problems. A manual and an automatic classification approach were defined to conduct this evaluation. In the manual approach, six experienced and less experienced otolaryngologists individually evaluated PVC and LVC in CE-NBI images of patients and classified them into benign and malignant groups. An updated version of the algorithm proposed in [17,18] with 24 features and four supervised classifiers has been used to classify CE-NBI images into benign and malignant groups. The results of the two approaches were compared in terms of classification sensitivity and specificity. The potential of an automatic approach to assist the clinicians is presented through two evaluation strategies.

## 2. Material and Methods

### 2.1. Data Acquisition

CE-NBI images were extracted from video scenes of adult patients who received a microlaryngoscopy for benign, pre-malignant or malignant lesions of the vocal folds. Video scenes were captured using an Evis Exera III Video System with integrated NBI-filter (Olympus Medical Systems, Hamburg, Germany) and a rigid 30-degree contact endoscope (Karl Storz, Tuttlingen, Germany) with a fixed magnification of 60 to have a fixed camera–tissue distance. For each video scene, we selected the time intervals where the video quality was good enough to visualize the vessels. Then, one in every ten frames was extracted from the selected intervals in JPEG format images (1008×1280 pixels) to have unique and non-redundant CE-NBI images.

### 2.2. Dataset Generation

The CE-NBI dataset included 1632 extracted images of 68 patients. The patients’ data were pseudonymized. Based on the WHO classification [19], histological diagnoses were used to label images as belonging to a benign or a malignant class. Table 1 shows the histopathologies with the number of patients and images used for the generation of the dataset.

**Figure 1:**
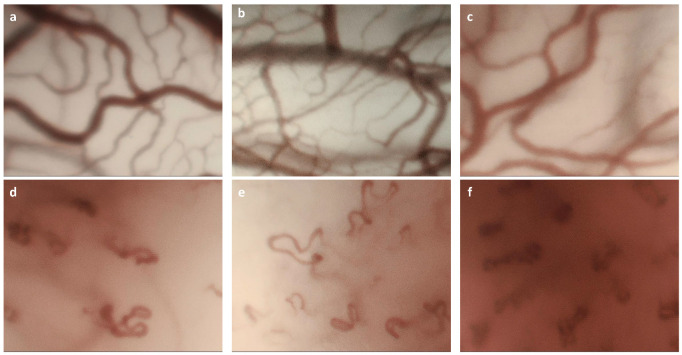
Examples of Longitudinal Vascular Changes (LVC) and Perpendicular Vascular Changes (PVC) in Contact Endoscopy (CE)-Narrow Band Imaging (NBI) images with different histopathologies: (**a**) Reinke’s edema, LVC; (**b**) polyp, LVC; (**c**) amyloidosis, LVC; (**d**) severe dysplasia, PVC, (**e**) carcinoma in situ, PVC; (**f**) Squamous Cell Carcinomas (SCC), PVC.

Two image subsets were created from the CE-NBI dataset. The *Subset I* included a series of two to five randomly selected CE-NBI images of each patient—total of 336 images, ≈20% of the dataset. The *Subset II* included the rest of the CE-NBI images—a total of 1296 images, ≈80% of the dataset, and was used as the training set of the automatic approach. The *Subset I* was evaluated by the otolaryngologists in the manual approach and then used as the testing set for the automatic approach. Figure 1 presents some examples of CE-NBI images with LVC and PVC belonging to the generated dataset.

### 2.3. Manual Approach

Three specialist and three resident otolaryngologists evaluated the images and classified the patients into benign and malignant groups. The residents had less than two years of experience in operating with CE-NBI images and the specialists worked for more than five years with such images. The otolaryngologists were blinded to the histologic diagnosis. They used the ELS guideline to independently visually evaluate the CE-NBI images of *Subset I* based on PVC appearance in the CE-NBI images, as explained in [12].

### 2.4. Automatic Approach

We used the algorithm presented in [17,18] to perform the automatic approach. The algorithm consists of a pre-processing step involving vessel enhancement and segmentation [20]. A feature extraction step was then applied to extract 24 geometrical features based on the consistency of gradient direction and the curvature level. Supervised classification step was conducted using the features and four classifiers to classify CE-NBI images into benign and malignant groups.

In this study, we made two main changes to the algorithm proposed in [17,18]. First, the Jerman filter [21] was used as pre-processing for the vessel enhancement step instead of the Frangi filter to overcome the problems related to the established enhancement function, not well adapted to natural variations of the vascular morphology. Second, the values of the tuning parameters of four classifiers including Support Vector Machine (SVM) with Polykernel and Radial Basis Function (RBF) [22], k-Nearest Neighbor (kNN) [23] and Random Forest Classifier (RFC) [24] were updated to have the optimum classification results with the current dataset.

In order to cover all the possible vascular structures, the vesselness parameter σ of the Jerman filter was set in the range of 0.5 mm to 2.5 mm with a step size of 0.5 mm. The parameter τ controlling the response uniformity was empirically set as 1.

The hyperparameter tuning process of all classifiers was updated using a grid search combined with 10-fold cross validation.

The performance of SVM is maily affected by the regulation parameter (*C*) and kernel parameter (γ). The regulation parameter together with Polykernel and RBF controls the trade-off between achieving a low error in training data. γ determines how quickly class boundaries dissipate when they get far from the support vectors in SVM with RBF. The range of *C* and γ values were set within the range of 0.001 to 1000 with a ten-fold increment. The SVM with RBF completed the high overall performance with C=1 and γ=0.01 and SVM with Polykernel indicated the best results with C=1.

Euclidean Distance was applied to calculate the distance of a sample in the case of kNN. To select the optimum *k*, a range from 1 to 20 with the step size equal to one were used. kNN confirmed the best performance at k=5.

The optimization for RFC was done by adjusting the depth of trees and the number of estimators. The range of depth of the trees was set from 1 to 20 with step size equal to one. For the number of estimators, values from 10 to 100 with an increase of five was defined. The classifier gave the best performance at a depth of 8 with 55 trees.

In all classification scenarios, Subset I and Subset II were used as the testing and training sets, respectively. CE-NBI images were labeled as 0 for benign and as 1 for malignant groups. Each classifier was trained using the images’ labels and feature vectors that were computed form the CE-NBI images of the training set. For the testing, the features vectors computed from the CE-NBI images of the testing set were fed into the predictive model of each classifier and then the expected labels were collected.

## 3. Evaluation Strategy

### 3.1. Classification Performances of Manual and Automatic Approaches

The global performances of the manual and automatic classification were evaluated using two classification measurements: sensitivity and specificity.

In the manual classification, the otolaryngologists assessed the set of CE-NBI images in the *Subset I* and classified each patient’s image set as benign or malignant. Following [12], the PVC-positive patients with the malignant histological diagnosis were considered as true positive cases. With this assumption, a confusion matrix was created and the average value of sensitivity and specificity of all otolaryngologists, specialists and residents, was calculated using the following parameters:

- True Positive: PVC-positive patients with malignant lesions.
- True Negative: PVC-negative patients with benign lesions.
- False Negative: PVC-negative patients with malignant lesions.
- False Positive: PVC-positive patients with benign lesions.

In the automatic classification, the classifiers classified each CE-NBI image of *Subset I* as benign or malignant. A confusion matrix was calculated for each classifier using the predicted and actual labels of the images. Then, sensitivity and specificity were calculated using the following parameters:

- True Positive: actual image label is malignant, predicted image label is malignant.
- True Negative: actual image label is benign, predicted image label is benign.
- False Negative: actual image label is malignant, predicted image label is benign.
- False Positive: actual image label is benign, predicted image label is malignant.

Based on the descriptions above, the sensitivity and specificity values can show the performances of classifiers/otolaryngologists to correctly classify malignant and benign images/patients.

### 3.2. Comparison Procedure between Manual and Automatic Classification

In a routine clinical procedure, the otolaryngologist evaluates a set of CE-NBI images of a patient and then identifies a patient’s lesion as benign or malignant. For the manual classification in this work, the clinicians performed a similar routine, making a decision based on a set of images belonging to a patient. Since the automatic classification does not classify a patient but an image, in order to compare automatic to manual classification we made the following assumption: if a given classifier correctly classifies more than half of the images of a patient, then the patient is considered as a correct classification performed by this classifier. Following the assumption, two procedures for comparing between manual and automatic classification were proposed.

The first comparison procedure consists of comparing both approaches based on the level of agreement/disagreement between clinicians for classifying a patient as benign or malignant. In this aim, patients were divided into three categories:

- Category I includes 29 patients. All otolaryngologists correctly classified these patients.
- Category II includes 26 patients. One to five otolaryngologists correctly classified these patients.
- Category III includes 13 patients. All otolaryngologists misclassified these patients.

The second comparison procedure aims to compare manual and automatic classifications in terms of their misclassification levels depending on the histopathologies. This evaluation was performed to analyze the histopathologies in benign and malignant groups that caused significant difficulties for otolaryngologists and then to see how the automatic approach behaves with these cases.

We divided the patients into the 15 groups presented in Table 1. For each histopathology, a misclassification percentage was computed per patient for the automatic and manual classification as follows:

- Misclassification percentage of all otolaryngologists per patient in each histopathology group:
  (1) $$\left(\frac{\mathrm{Number}\;\mathrm{of}\;\mathrm{doctor}(s)\;\mathrm{who}\;\mathrm{misclassified}\;\mathrm{the}\;\mathrm{patients}}{\mathrm{Total}\;\mathrm{number}\;\mathrm{of}\;\mathrm{doctors}\times \mathrm{Total}\;\mathrm{number}\;\mathrm{of}\;\mathrm{patients}}\right)\times 100$$
  where the total number of patients was the number of patients for the corresponding histopathology.
- Misclassification percentage of every classifier per patient in each histopathology group:
  (2) $$\left(\frac{\mathrm{Number}\;\mathrm{of}\;\mathrm{misclassified}\;\mathrm{patient}(s)}{\mathrm{Total}\;\mathrm{number}\;\mathrm{of}\;\mathrm{patients}}\right)\times 100$$
- Misclassification percentage of all classifiers per patient in each histopathology group:
  (3) $$\left(\frac{\mathrm{Number}\;\mathrm{of}\;\mathrm{misclassified}\;\mathrm{patient}(s)\;\mathrm{by}\;\mathrm{all}\;\mathrm{classifiers}}{\mathrm{Total}\;\mathrm{number}\;\mathrm{of}\;\mathrm{classifiers}\times \mathrm{Total}\;\mathrm{number}\;\mathrm{of}\;\mathrm{patients}\;}\right)\times 100$$

## 4. Results and Discussion

Table 2 shows the global performances of the manual and automatic classification. In the manual approach, otolaryngology specialists showed a better performance than the otolaryngology residents. These results prove that the interpretation of CE-NBI images based on vascular patterns is subjective and highly depends on otolaryngologists’ experience.

For the automatic approach, RFC with a sensitivity of 0.846 and SVM with RBF kernel with a specificity of 0.981 showed better results in comparison to the other classifiers.

The overall specificity values of otolaryngologists are low. This means that both groups had difficulties in distinguishing patients with benign histopathologies from malignant ones visually. This fact can be explained by the similarity between vascular patterns of benign and malignant histopathologies that can not be distinguished easily. For instance, Papillomatosis is a benign histopathology with similar vascular patterns than malignant histopathologies. This similarity leads to visually misclassify Papillomatosis as malignant. However, all four classifiers showed higher specificity than otolaryngologists proving the ability of automatic approach to overcome such a problem.

**Figure 2:**
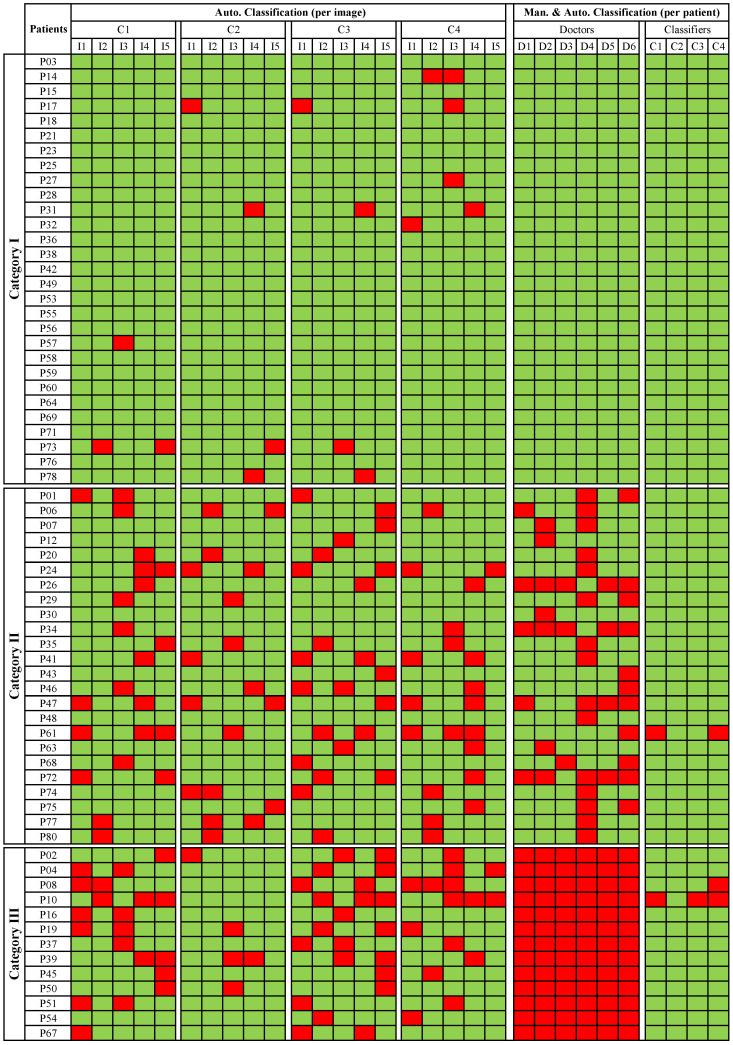
An overall view of the manual and automatic classification of every patient of the dataset; Green color: correct classification; Red color: misclassification. C1 to C4 represents the four classifiers; C1: Support Vector Machine (SVM) with polykernel, C2: SVM with Radial Basis Function (RBF), C3: k-Nearest Neighbor (kNN) and C4: Random Forest Classifier (RFC). I1 to I5 represent five testing images for each patient. D1 to D6 represent the six otolaryngologists.

Figure 2 shows the detailed results of the first comparison procedure consisting of comparing both approaches based on the level of agreement/disagreement between clinicians for classifying a patient as benign or malignant. A first visual inspection shows that the classifiers individually misclassified 1 to 2 images in some patients at the Category I, where all otolaryngologists correctly classified these patients. Nevertheless, based on the assumption made in Section 3.2, the automatic approach did not misclassify any patient of this category.

**Figure 3:**
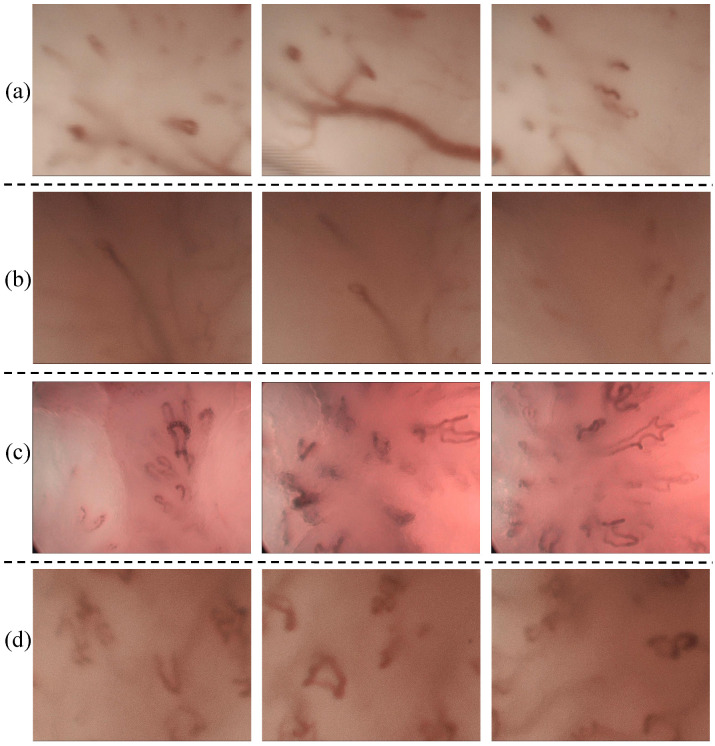
CE-NBI images of four patients from Category II and Category III: (**a**) p26, (**b**) p34, (**c**) p72 and (**d**) p10.

For the patients belonging to Category II, both manual and automatic increased their misclassification levels compared to Category I. In the automatic approach, it is possible to observe that several images belonging to a patient can be misclassified. However, if we consider the automatic classification per patient, only for one patient, two classifiers (SVM with polykernel and RFC) perform a misclassification. On the other hand, otolaryngologists showed a significant misclassification in some cases. For example, in the case of patients p26, p34 and p 72, five clinicians misclassified the patients, while the classifiers classified the patients correctly. These patients were diagnosed as Papillomatosis and Hyperkeratosis cases and belong to benign histopathologies. Figure 3a–c, displays the PVC vascular patterns in the CE-NBI images of these patients. As pointed out in the introduction, the difference between PVC in benign and in malignant histopathologies is not visually evident for the otolaryngologist. This causes a significant difficulty for the clinicians to distinguish benign from malignant cases based on the vascular patterns. Based on the results, the automatic approach showed the ability to identify this difference and then classify the patients correctly because it is capable of quantifying and differentiating these tiny differences. SVM with RBF did not show any misclassification per patient in this category.

For the Category III, where all otolaryngologists misclassified the patients, SVM with RBF misclassified fewer images compared to the other three classifiers. Concerning the classification per patient performed by the classifiers, it is possible to see that misclassifications were made for only two patients. Particularly, for patient p10 three classifiers failed in their classification. According to the histopathology, it corresponds to a patient presenting Hyperkeratosis. A set of CE-NBI images of this case is presented in Figure 3d. The type of vascular patterns of Hyperkeratosis can notably vary from one patient to another one. The CE-NBI dataset included 4 patients for this histopathology, presenting LVC and PVC vascular patterns. Due to this variation, the classifier’s learning process using the proposed features [17,18] can be complicated. SVM with RBF showed no misclassification per patient in this Category.

These results show that the complexity of a manual analysis of a laryngeal lesion can be related to the type of histopathology and therefore we decided to perform a separated analysis based on the histopathology of the lesion. Table 3 presents the results of this second comparison procedure.

For the benign histopathologies, otolaryngologists showed high misclassification percentage of 83%, 77%, 46%, 33% and 27% for Fibroma, Papillomatosis, Hyperkeratosis, Squamous Hyperplasia and Polyp, respectively. Except for Fibroma, the misclassification level of each classifier is lower than the manual classification. Notably, in the case of Papillomatosis, the misclassification is significantly reduced in each classifier. If all classifiers are considered, the misclassification decreases from 77% to 7% in this histopathology. Papillomatosis causes classification difficulties to the otolaryngologists due to their vascular patterns that has similar characteristics to the malignant histopathologies. SVM with RBF and kNN seems to have the ability to solve this issue with 0% misclassification.

In the case of Fibroma, the misclassification percentage varied significantly among the four classifiers. This can be explained by the reduced number of images that the dataset contains for this type of histopathology (only one patient and two images).

In the malignant group, the otolaryngologists had the highest misclassification percentage of 61% for mild dysplasia. This histopathology can have PVC as well as LVC vascular patterns that usually appear in benign histopathologies. Hence, it is challenging for the otolaryngologists to classify patients with this condition as malignant visually. For this histopathology, the four classifiers performed well by classifying every patient correctly.

In general, SVM with RBF showed no patient misclassification for all histopathologies.

## 5. Conclusions

Assessment of vascular patterns in CE-NBI images of vocal folds can provide valuable information for the clinicians to make the correct diagnostic decision before treatment. In this study, we showed how the evaluation of vascular patterns can be challenging for the otolaryngologists and how a computer-based approach can help clinicians ease this process.

In general, the otolaryngology specialists showed better classification performance than the residents in the manual approach. This proves that the interpretation of vascular patterns is subjective and depends on the clinicians’ experience, as pointed out by several publications [4,10,12,15,16]. Both groups of otolaryngologists showed relatively low specificity on classifying a case as benign or malignant. This explains the difficulties in the visual classification of benign histopathologies. In the case of the benign group, otolaryngologists had the highest misclassification percentage for Papillomatosis and Hyperkeratosis. In the automatic approach, all four classifiers showed a higher specificity than both groups of otolaryngologists and showed significantly less misclassification percentage for Papillomatosis and Hyperkeratosis. The otolaryngology specialists showed significantly higher sensitivity than the residents. This means that specialists with more experience can easily detect PVC in CE-NBI images, while it is more challenging for the residents. In the malignant group, most of the misclassifications of otolaryngologists happened in the case of Mild Dysplasia and SCC. Although all classifiers showed lower sensitivity than otolaryngology specialists, they significantly reduced the misclassification percentage for Mild Dysplasia and SCC, compared to the otolaryngologists.

Two facts can explain the lower sensitivity and higher misclassification percentage that the classifiers show in the malignant group than the benign group. First, the CE-NBI dataset included more images in the benign group than in the malignant group (less training images were available for the malignant group). A significant part of CE-NBI images of the benign group belonged to the Papillomatosis with PVC patterns similar to those of malignant histopathologies. Second, the 24 features take only into account geometrical characteristics of the vascular patterns and no other characteristics that can also be important for the classification procedure. Due to these two points, it is possible that the algorithm shows some errors and classifies the CE-NBI images of malignant cases as benign. Hence, it is important to balance the number of CE-NBI images in the dataset for future works and develop new methods to improve the differentiation between wide and narrow angled points of PVCs.

The automatic approach showed its capacity to perform as an assistant system when there are disagreements among otolaryngologists or when they all misclassified the patients. SVM with RBF had the best performance and did not show any misclassification per patient in all the categories. This means that the combination of the proposed 24 features and SVM with RBF classifier, can provide valuable feedback for the clinicians to make decisions regarding the treatment planning. In general, the automatic approach has the potential to overcome the current issues in the field of enhanced CE and can operate as an assisting system to provide a more confident way for clinicians to learn as well as to make intraoperative decisions about the method and extent of surgical resection in patients with laryngeal cancer or benign vocal fold lesions in the routine surgical procedures.

## Figures and Tables

**Table 1:** Histopathologies used for the generation of the dataset.

Type of Lesion	Histopathology	Number of Patients	Number of Images
**Benign**	Cyst	3	90
	Polyp	5	71
	Reinke’s edema	12	329
	Hyperkeratosis	4	82
	Squamous Hyperplasia	3	75
	Papillomatosis	11	286
	Amyloidosis	2	32
	Nodule	1	26
	Granuloma	1	28
	Fibroma	1	2
**(Pre)Malignant**	Mild Dysplasia	3	77
	Moderate Dysplasia	2	49
	Severe Dysplasia	3	68
	Carcinoma In Situ	9	249
	SCC	8	168
**Total**		**68**	**1632**

**Table 2:** General performance of manual and automatic approaches.

Classification Measurements		Sensitivity	Specificity
**Manual Classification (per patient)**	Otolaryngology specialists	0.955	0.727
	Otolaryngology residents	0.630	0.609
	All otolaryngologists	0.818	0.630
**Automatic Classification (per image)**	SVM with polykernel	0.830	0.882
	SVM with RBF	0.806	0.981
	kNN	0.814	0.863
	RFC	0.846	0.895

**Table 3:** Misclassification percentage of every histopathology category based on patient. C1 to C4 represent the four classifiers; C1: SVM with polykernel, C2: SVM with RBF, C3: kNN and C4: RFC.

Type of Lesions	Histopathology	Man. and Auto. Classification (per Patient)					
		Doctors	C1	C2	C3	C4	All Classifiers
**Benign**	Cyst	0%	0%	0%	0%	0%	0%
	Polyp	27%	0%	0%	0%	0%	0%
	Reinke’s edema	7%	0%	0%	0%	0%	0%
	Hyperkeratosis	46%	25%	0%	25%	25%	19%
	Squamous Hyperplasia	33%	0%	0%	0%	0%	0%
	Papillomatosis	77%	9%	0%	0%	18%	7%
	Nodule	0%	0%	0%	0%	0%	0%
	Granuloma	0%	0%	0%	0%	0%	0%
	Amyloidosis	8%	0%	0%	0%	0%	0%
	Fibroma	83%	0%	0%	100%	100%	50%
**(Pre)Malignant**	Mild Dysplasia	61%	0%	0%	0%	0%	0%
	Moderate Dysplasia	17%	0%	0%	0%	0%	0%
	Severe Dysplasia	17%	0%	0%	0%	0%	0%
	Carcinoma In Situ	9%	0%	0%	0%	0%	0%
	SCC	25%	0%	0%	13%	0%	3%
